# Nivolumab plus Carboplatin and Paclitaxel as the First-line Therapy for Advanced Squamous Cell Carcinoma of the Lung with Strong Programmed Death-ligand 1 Expression: A Case Report

**DOI:** 10.7759/cureus.5881

**Published:** 2019-10-10

**Authors:** Frank S Fan, Chung-Fan Yang, Chia-Lin Chang

**Affiliations:** 1 Haematology and Oncology, Changhua Hospital, Ministry of Health and Welfare, Chang-Hua County, TWN; 2 Pathology, Changhua Hospital, Ministry of Health and Welfare, Chang-Hua County, TWN; 3 Haematology and Oncology, Feng-Yuan Hospital, Ministry of Health and Welfare, Taichung City, TWN

**Keywords:** nivolumab, chemotherapy, squamous non-small cell lung cancer, immune checkpoint inhibitor, pneumonitis

## Abstract

An 80-year-old male patient was diagnosed to have squamous cell carcinoma of the lung which had a high level of programmed death-ligand 1 (PD-L1) expression. He was prescribed with intravenously administered nivolumab combined with carboplatin and paclitaxel as the first-line therapy. A rapid remission was achieved with nearly total necrosis and cavitation of the original tumor. However, the successful treatment result was accompanied with pneumonitis most likely as an adverse effect of nivolumab. After discontinuation of nivolumab and starting prednisolone treatment, the pneumonitis was soon brought under control. During the treatment course, temporary exacerbation of the disease status led to an interesting differential diagnosis between hyperprogression and pseudoprogression. Tremendous efficacy of combination immunochemotherapy as the first-line treatment for squamous non-small cell lung cancer (NSCLC) with highly expressed PD-L1 has been well demonstrated in this case.

## Introduction

Immune checkpoint inhibitors (ICI), including anti-programmed death 1 (PD-1) and anti-programmed death-ligand 1 (PD-L1) antibodies, have evolved to be the most hopeful and attractive therapeutic modalities for non-small cell lung cancer (NSCLC) without targetable genetic mutations [1]. Theoretically, ICI treatment effects could be potentiated by tumor antigen release during traditional systemic chemotherapy and this assumption has led to the development of combining ICI and chemotherapy together as a new anti-cancer strategy [2]. Accordingly, several clinical trials testing this hypothesis have been designed to see whether combination therapy as first-line treatment could achieve better survival than chemotherapy alone in advanced NSCLC [3].

Nivolumab, an anti-PD-1 antibody, combined with platinum doublet chemotherapy, has shown satisfactory tumor size reduction and response duration for chemotherapy-naïve advanced NSCLC in two phase I clinical trials [4-5]. In the phase III trial CheckMate 227 Part 2, although nivolumab plus chemotherapy versus chemotherapy alone did not meet its primary endpoint of overall survival in first-line treatment for non-squamous NSCLC, an exploratory analysis of patients with first-line treatment for squamous NSCLC disclosed that the median overall survival was 18.27 months for nivolumab plus chemotherapy versus 11.96 months for chemotherapy with a hazard ratio of 0.69 and 95% confidence interval 0.50-0.97 [6]. 

Herein, we present the clinical course, astonish efficacy, and ICI-related pneumonitis in an inoperable squamous NSCLC patient receiving nivolumab and carboplatin plus paclitaxel as the first-line treatment.

## Case presentation

An 80-year-old male patient appeared in our clinic with the primary complaint of low back pain for one month and progressive weakness for one week in February 2019. Frequent dry cough lasting for about six weeks was also noted. He took daily low-dose aspirin and underwent occasional therapeutic phlebotomy for polycythemia vera, an inherited condition in his family without identifiable *JAK2* gene variants in the past five years. Besides, he had been on regular medical control for hyperlipidemia and benign prostate hyperplasia. His surgical history included a right nephrectomy for lesions of unknown nature and fixation for left clavicle fracture more than 10 years ago. He is a cigarette smoker, consuming half a pack per day for about 60 years.

On physical examination, there was no superficial lymphadenopathy and other remarkable findings except for coarse breathing sound over the left upper lung area and a certain tenderness over the lumbar spine. Mild elevation of fasting blood sugar (122 mg/dl) was noted upon laboratory examination. Serum carcinoembryonic antigen, cancer antigen 19-9, prostate-specific antigen, and renal and liver function were within normal limits. There was no evidence of viral hepatitis B and C infection. His blood routine showed slight leukocytosis (white blood cell 12,500/ml, neutrophil 81.5%), rather than normal thrombocyte count (314,000/ml), hemoglobin level (14.9 gm/dl), and hematocrit (45.1%).

**Figure 1:**
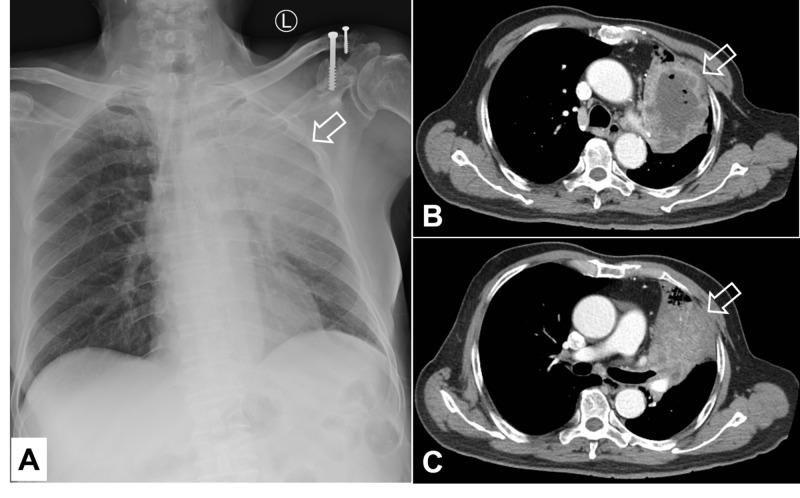
Radiological findings of a massive space-occupying lesion in the upper lobe of the lung at presentation: A. chest X-ray film, B and C. CT scan Arrows: Targets of interest

Magnetic resonance imaging revealed a degenerative thoracic spine and a compression fracture of the lumbar 1 vertebral body without evidence of metastasis. Chest X-ray, however, disclosed a massive space-occupying opacity over the left upper lung field (Figure 1A). Computed tomography (CT) scan showed an approximately 10-cm mass with central low attenuation and gas in the left upper lobe, abutting the left pulmonary artery and pleura with contrast enhancement of the solid portions (Figures 1B, 1C). 

**Figure 2:**
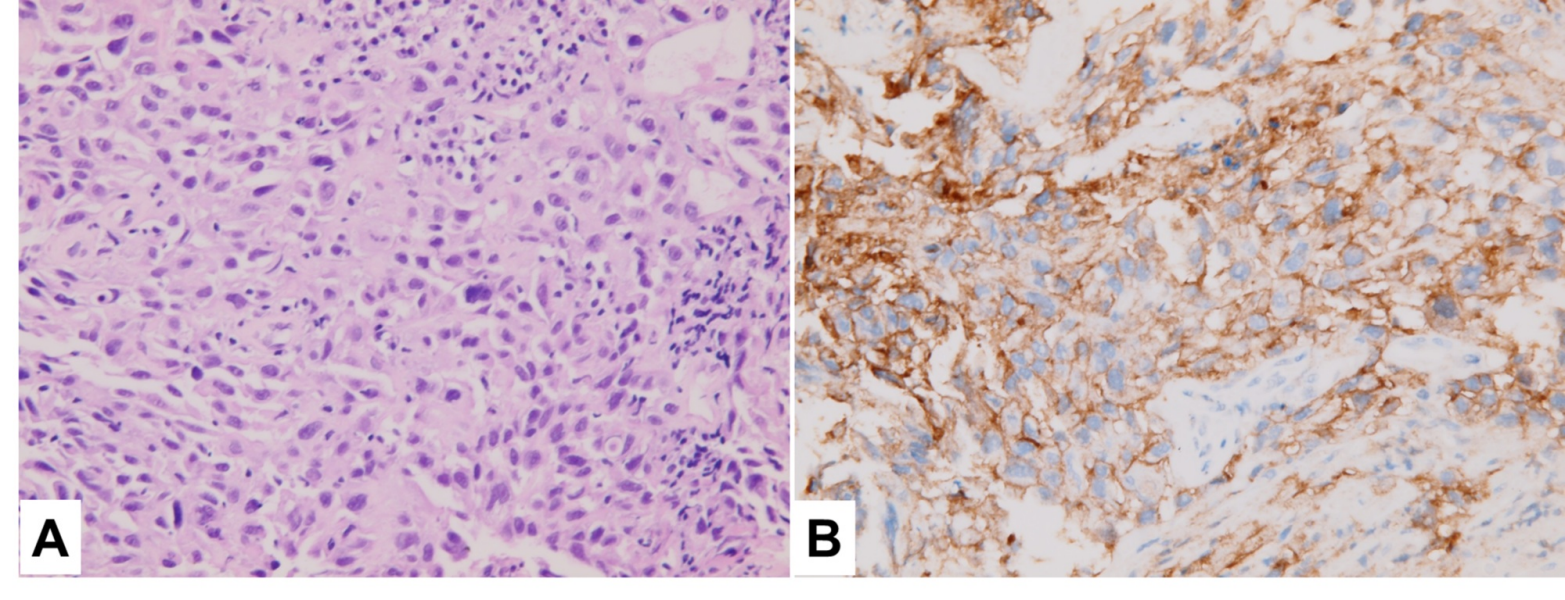
Poorly differentiated squamous non-small cell carcinoma of the lung, left upper lobe, bronchoscopy biopsy: A. Hematoxylin and eosin stain x400, B. PD-L1 strongly positive on immunohistochemical stain with tumor proportion score over 50%

Subsequent bronchoscopy brushing cytology and biopsy from an occlusive lesion in the left upper lobe bronchus led to a diagnosis of poorly differentiated carcinoma positive to p40 (+++) and CK5/6(+++), negative to CK7, TTF-1, and Napsin-A immunostains, in favor of squamous cell carcinoma (Figure 2A). Strongly positive PD-L1 expression was found in immunohistochemical staining with DAKO monoclonal rabbit anti-PD-L1 antibody clone 28-8 (Figure 2B). The tumor proportion score category for PD-L1 was ≥50%. 

**Figure 3:**
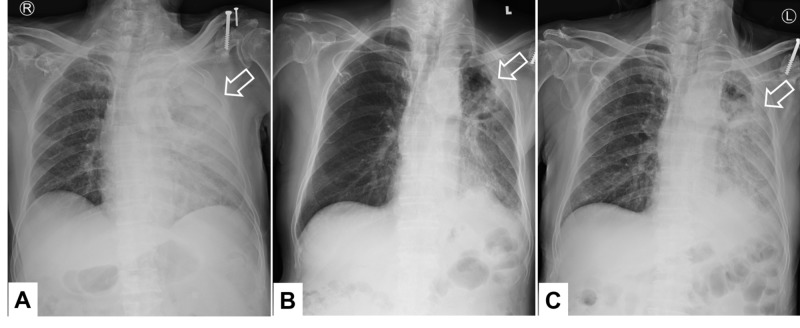
Chest X-ray films along the clinical course: A. March 19, 2019, B. April 30, 2019, C. May 13, 2019 Arrows: Targets of interest

The patient’s daughter and son brought the patient to another hospital for second opinion consultation. Chest medicine specialists in that hospital gave a suggestion of hospice care only which was unacceptable to them. We decided to treat him with carboplatin (target area under the curve 5) and paclitaxel (175 mg/m2) every four weeks as the first-line therapy in March 2019. On the request of the patient’s family, nivolumab (3 mg/kg every two weeks) was added ever since the second course of chemotherapy. After two doses of both nivolumab and carboplatin plus paclitaxel, dramatic response was detected on chest radiographs (Figures 3A, 3B). Nonetheless, the pulmonary opacity rebounded two weeks later after one more dose of nivolumab (Figure 3C). This warning change was considered to be either pseuoprogression or hyperprogression phenomenon.

**Figure 4:**
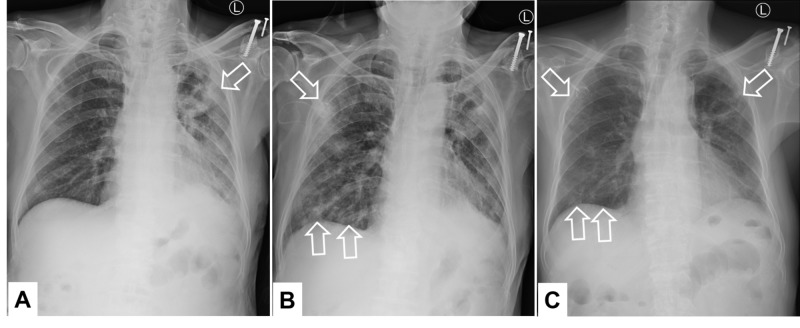
Chest X-ray films along the clinical course: A. May 29, 2019. B. June 10, 2019. C. June 20, 2019 Arrows: Targets of interest

Fortunately, hyperprogression was thought to be ruled out based on improvement of chest image after the fourth course of nivolumab and carboplatin plus paclitaxel (Figure 4A). Nevertheless, he complained of mild chest discomfort and multiple strange dense shadows appeared in the chest X-ray film shortly after the fifth dose of nivolumab, suggesting nivolumab-induced pneumonitis (Figure 4B). These new lesions resolved to a great extent following a further course of nivolumab plus carboplatin and paclitaxel therapy along with oral prednisolone (1 mg/kg) in eight days (Figure 4C). There was neither respiratory distress nor the need for oxygen inhalation support up to this point. 

**Figure 5:**
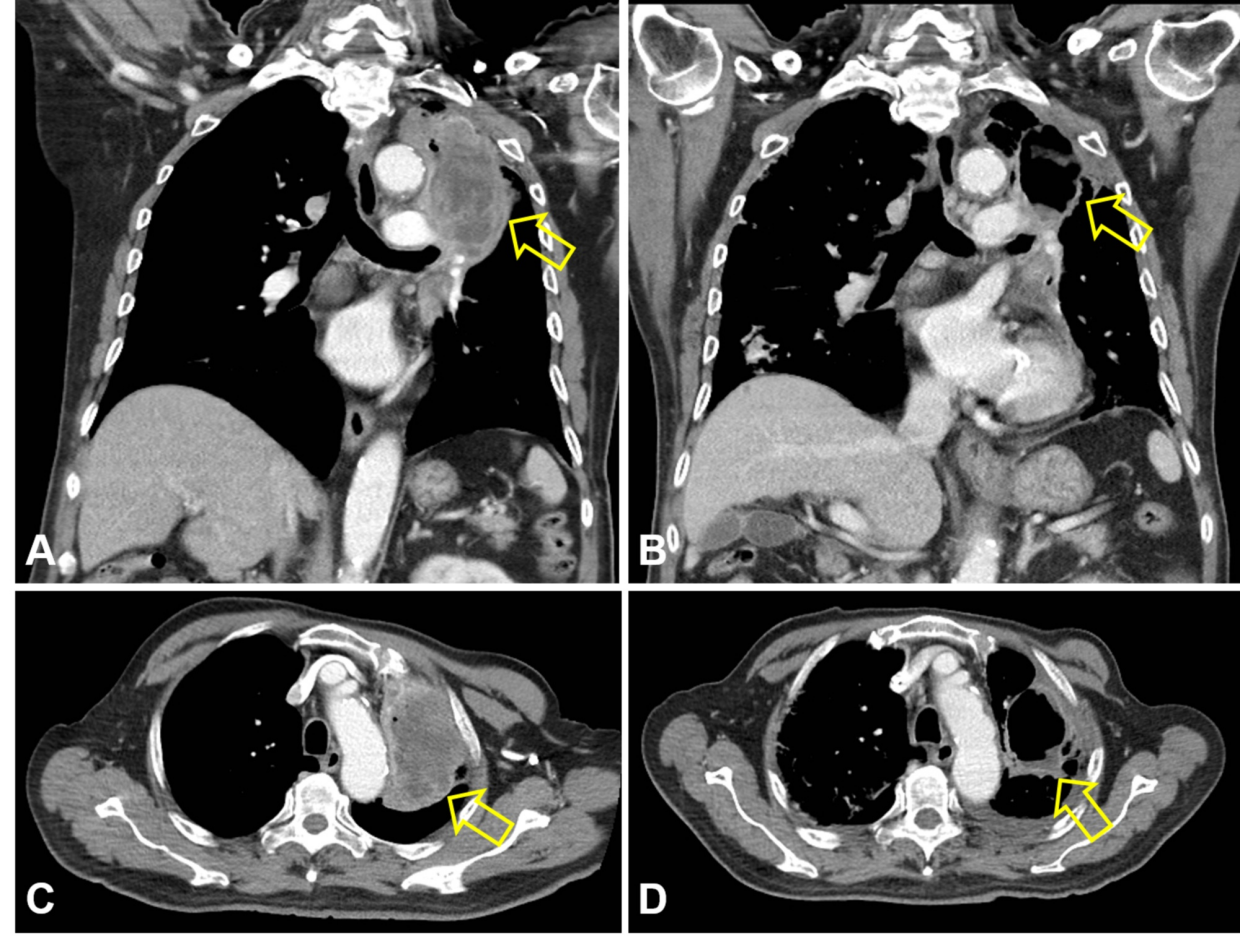
CT scan showing nearly complete necrosis and cavitation of the tumor after therapy: A and C. February 21, 2019. B and D. June 12, 2019 Arrows: Targets of interest CT, computed tomography

**Figure 6:**
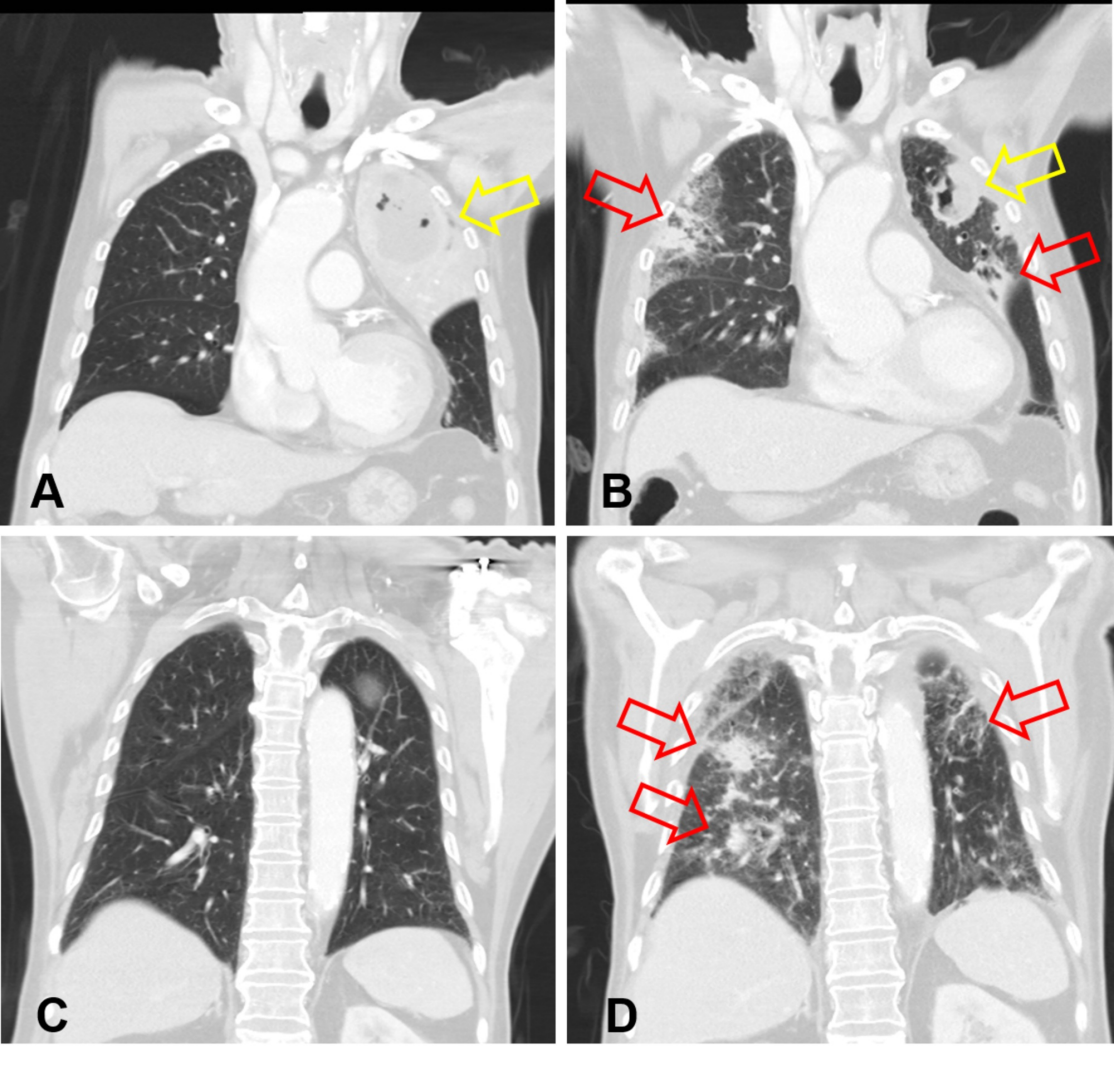
CT scan (lung window) disclosing development of pneumonitis (red arrows) along with tumor necrosis (yellow arrows) after therapy: A and C. February 21, 2019. B and D. June 12, 2019

**Figure 7:**
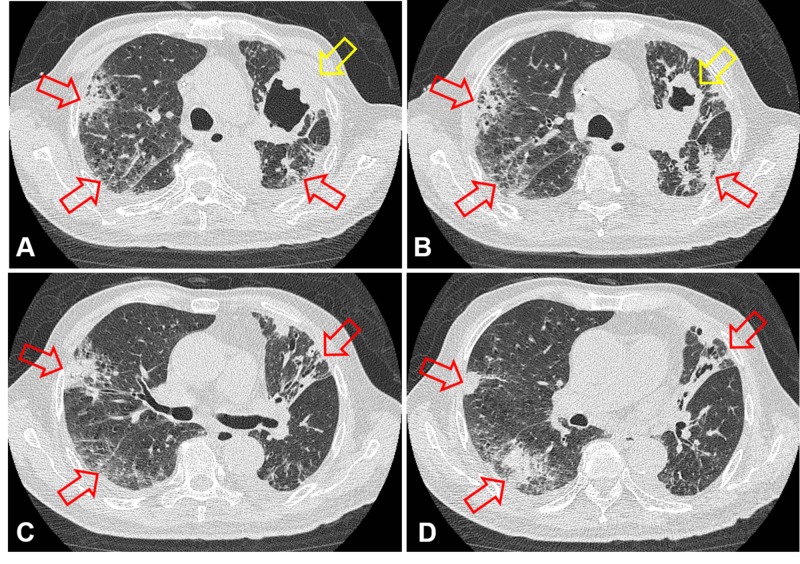
Tumor cavitation (yellow arrows) and multiple foci of pneumonitis (red arrows) on sequential axial CT scan (lung window): A to D. June 12, 2019

CT scan performed at the appearance of suspect pneumonitis confirmed the diagnosis. Although astonish tumor necrosis and cavitation were achieved as compared with scans taken prior to combined immunochemotherapy (Figures 5A-5D), newly developed cryptogenic organizing pneumonia, a form of idiopathic interstitial pneumonia, over both the lung fields could be clearly observed (Figures 6A-6D, 7A-7D). 

**Figure 8:**
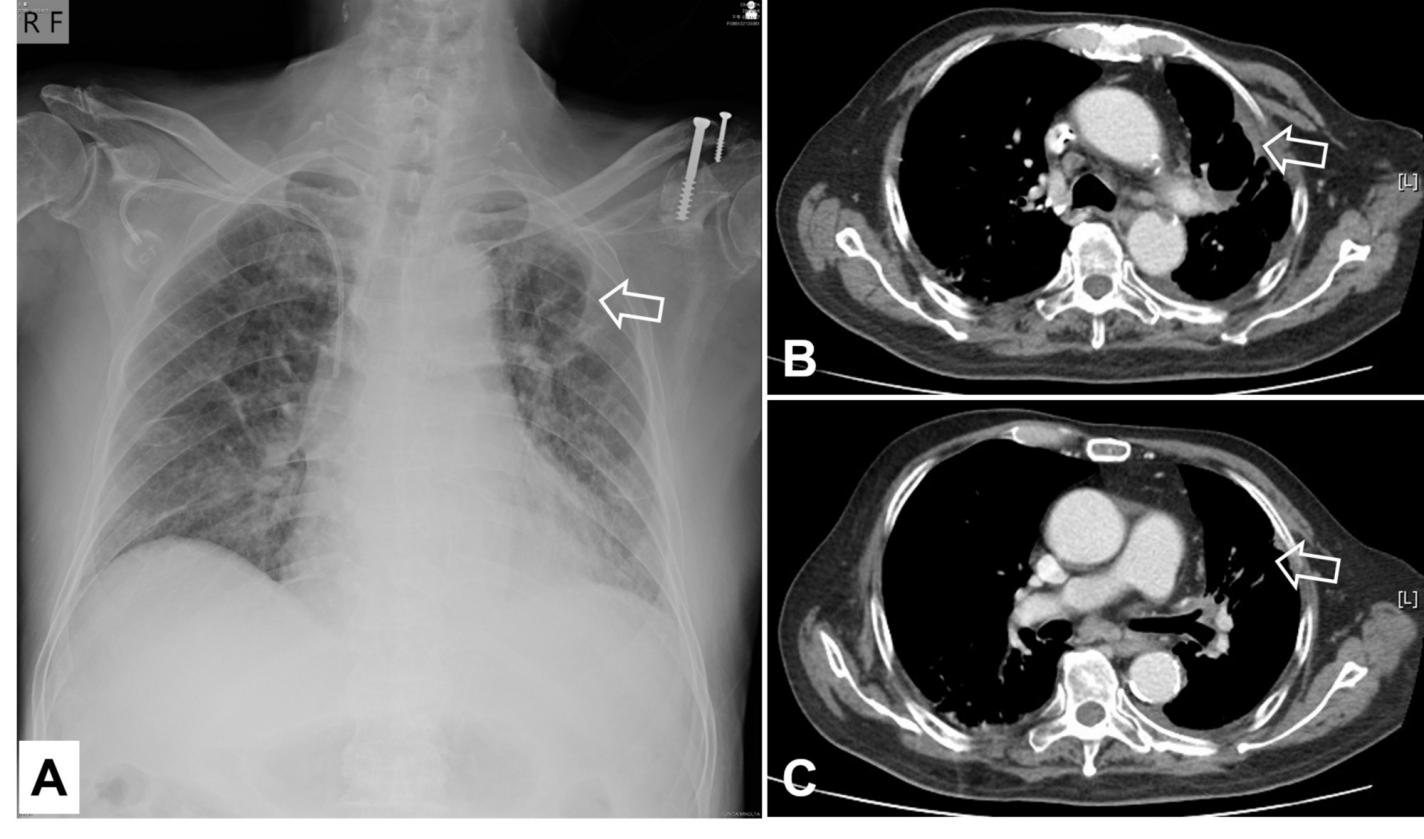
Radiological findings of nearly complete remission of the original malignant lesion, as compared with Figure 1: A. Chest X-ray film, September 6, 2019; B and C. CT scan, September 9, 2019 Arrows: Targets of interest

**Figure 9:**
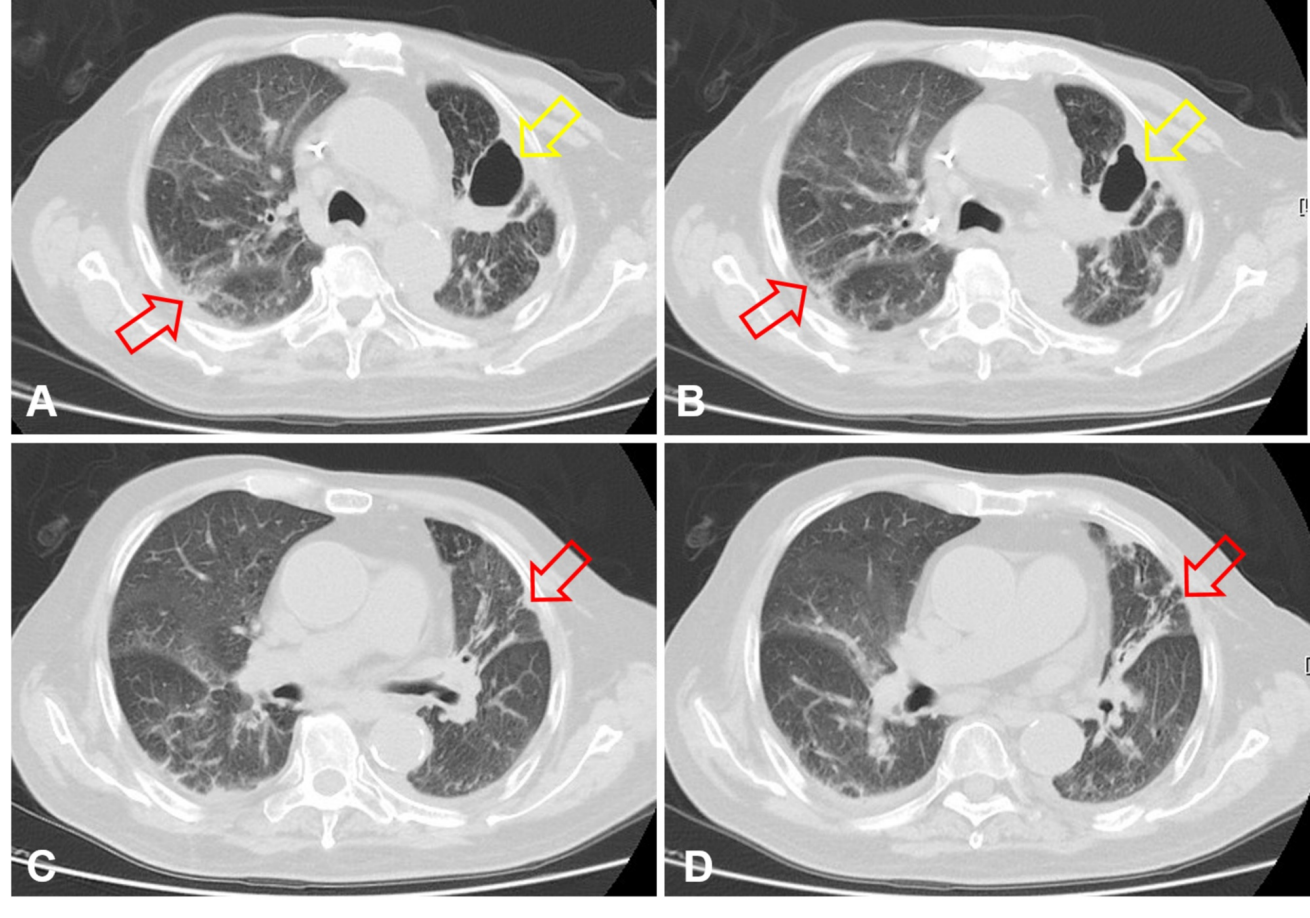
Progressively shrunken tumor cavity (yellow arrows) and much improvement of the pneumonitis with residual fibrotic changes (red arrows) on sequential axial CT scan (lung window) as compared with Figure 7: A to D, September 9, 2019

Nivolumab was discontinued after the sixth dose to avoid pneumonitis progression. Carboplatin and paclitaxel were given up to a total of five courses. Prednisolone therapy continued with the same dose for six weeks and then was shifted to a tapering schedule. Despite satisfactory tumor control has been maintained so far (Figures 8A-8C), severity of pneumonitis exacerbated and oxygen support became necessary upon a very low dose of prednisolone. His symptoms resolved when a prednisolone dose was included again. While preparing this case report, more than two months after stopping all immunochemotherapy, the patient has to continue the intake of prednisolone 10 mg twice daily for maintaining proper pulmonary function. At follow-up, CT scan showed progressive shrunken size of the tumor cavity and much improvement of the pneumonitis with some residual bronchiectasis-like pattern (Figures 9A-9D). 

During the whole course, there were no skin rash, diarrhea, abdomen pain, cardiac attack, neurologic deficit, renal insufficiency, liver function impairment, and thyroid and adrenal function abnormalities. Febrile neutropenia was noted once after the first course of chemotherapy. Adequate granulocyte-stimulating-factor prophylaxis was given since the second course of chemotherapy and the post-chemotherapy pancytopenia was generally tolerable thereafter.

## Discussion

A recent systemic review and meta-analysis of published clinical trials identified PD-L1 immunohistochemistry, tumor mutation burden, and gene expression profile to be the most valuable biomarkers for predicting tumor response to anti-PD-1/PD-L1 therapy [7]. A real-world study also showed that tumor PD-L1 expression correlated very well with clinical outcomes in NSCLC treated with anti-PD-1 antibody nivolumab and pembrolizumab [8]. Our patient’s high PD-L1 score and distinguished efficacy of nivolumab treatment are thus very compatible with these reports, supporting PD-L1 expression as a reliable biomarker for anti-PD-1 therapy in NSCLC.

In spite of the good therapeutic result, a short period of rebound opacity in chest film during the patient’s immunochemotherapy led to a difficult differential diagnosis between hyperprogression and pseudoprogression [9-10]. The fact that the opacity soon resolved on continuous nivolumab therapy made pseudoprogression a more likely diagnosis. However, the reverse of hyperprogression due to suppression of regulatory T cell (Treg) activity by simultaneous chemotherapy remained an interesting possibility [11]. If the latter presumption is true, a combination of ICI and chemotherapy might turn out to be a more reasonable choice against squamous cell lung cancer in the future.

As known, autoimmune adverse effects of ICI could almost take place in all organs over the body. The incidence of pneumonitis is about 3% to 8% [12]. The patterns of nivolumab-related interstitial lung disease in NSCLC mainly included acute interstitial pneumonia with diffuse alveolar damage, cryptogenic organizing pneumonia, hypersensitivity pneumonitis, and nonspecific interstitial pneumonia [13]. The initial management of ICI-related pneumonitis includes holding ICI agents immediately and starting prednisolone 1-2 mg/kg for at least four weeks before tapering slowly. Other immunosuppressive therapy such as infliximab, mycophenolate mofetil, intravenous immunoglobulin, and cyclophosphamide should be added if the pneumonitis status worsens out of control [14]. 

Our patient’s pneumonitis improved rapidly on recommended full dose prednisolone, but apparently, complete withdrawal of steroid is difficult if the underlying autoimmune process is still active. We do consider adopting additional immunosuppressive agents when necessary. On the other hand, in nivolumab-treated NSCLC, the occurrence of immune-related adverse events was found to be associated with better clinical outcomes, and severe nivolumab-induced pneumonitis leading to durable remission in a squamous NSCLC patient had been reported [15-16]. Hence, our patient would enjoy a rather good prognosis with regard to his lung cancer.

The question of whether durable remission could be maintained after stopping ICI similar to our case necessitates further studies; however, two recent case series have yielded positive results. One of them even concluded that durable response could be achieved after discontinuation of ICI in the absence of toxicity [17-18]. This point of view shall be appreciated by patients who develop intolerable adverse effects or lack enough financial support for ceaseless ICI treatment.

The U.S. Food and Drug Administration has published a meta-analysis of randomized NSCLC clinical trials, revealing that advanced NSCLC patients over 75 years of age seem to gain survival benefits from anti-PD-1/PD-L1 antibodies similar to patients younger than 65 years of age [19]. This finding should be very encouraging to our 80-year-old patient. Finally, the patient might also find some comfort that he had been a long-term smoker because, in comparison with chemotherapy, ICI treatment in NSCLC trials improved survival significantly in ever-smokers but not in never-smokers according to a recent analysis [20]. Of course, more investigations are required to confirm this result.

## Conclusions

Combination of nivolumab and carboplatin plus paclitaxel as the first-line therapy successfully brought a PD-L1-strongly-positive advanced squamous NSCLC in an elder patient into remission with the price of controllable ICI-related pneumonitis. Adverse effects of ICI may be awful but correlate with better response and longer survival. Concurrent chemotherapy not only destroys tumor cells and promotes tumor antigen release and presentation to immune cells, but could also contribute immensely to the suppression of Treg activity that plays a major role in hyperprogression under ICI therapy. We look forward to considering ICI combined with chemotherapy as the mainstay treatment for squamous NSCLC with high PD-L1 expression in the future. 
